# Forecasted Size of Measles Outbreaks Associated With Vaccination Exemptions for Schoolchildren

**DOI:** 10.1001/jamanetworkopen.2019.9768

**Published:** 2019-08-21

**Authors:** David R. Sinclair, John J. Grefenstette, Mary G. Krauland, David D. Galloway, Robert J. Frankeny, Clayton Travis, Donald S. Burke, Mark S. Roberts

**Affiliations:** 1 Graduate School of Public Health, University of Pittsburgh, Pittsburgh, Pennsylvania; 2 Texas Pediatric Society, the Texas Chapter of the American Academy of Pediatrics, Austin

## Abstract

**Importance:**

Vaccine exemptions, which allow unvaccinated children to attend school, have increased by a factor of 28 since 2003 in Texas. Geographic clustering of unvaccinated children facilitates the spread of measles introductions, but the potential size of outbreaks is unclear.

**Objective:**

To forecast the range of measles outbreak sizes in each metropolitan area of Texas at 2018 and future reduced school vaccination rates.

**Design, Setting, and Participants:**

An agent-based decision analytical model using a synthetic population of Texas, derived from the 2010 US Census, was used to simulate measles transmission in the Texas population. Real schools were represented in the simulations, and the 2018 vaccination rate of each real school was applied to a simulated hypothetical equivalent. Single cases of measles were introduced, daily activities and interactions were modeled for each population member, and the number of infections over the course of 9 months was counted for 1000 simulated runs in each Texas metropolitan area.

**Interventions:**

To determine the outcomes of further decreases in vaccination coverage, additional simulations were performed with vaccination rates reduced by 1% to 10% in schools with populations that are currently undervaccinated.

**Main Outcomes and Measures:**

Expected distributions of outbreak sizes in each metropolitan area of Texas at 2018 and reduced vaccination rates.

**Results:**

At 2018 vaccination rates, the median number of cases in large metropolitan areas was typically small, ranging from 1 to 3 cases, which is consistent with outbreaks in Texas 2006 to 2017. However, the upper limit of the distribution of plausible outbreaks (the 95th percentile, associated with 1 in 20 measles introductions) exceeded 400 cases in both the Austin and Dallas metropolitan areas, similar to the largest US outbreaks since measles was eliminated in 2000. Decreases in vaccination rates in schools with undervaccinated populations in 2018 were associated with exponential increases in the potential size of outbreaks: a 5% decrease in vaccination rate was associated with a 40% to 4000% increase in potential outbreak size, depending on the metropolitan area. A mean (SD) of 64% (11%) of cases occurred in students for whom a vaccine had been refused, but a mean (SD) of 36% (11%) occurred in others (ie, bystanders).

**Conclusions and Relevance:**

This study suggests that vaccination rates in some Texas schools are currently low enough to allow large measles outbreaks. Further decreases are associated with dramatic increases in the probability of large outbreaks. Limiting vaccine exemptions could be associated with a decrease in the risk of large measles outbreaks.

## Key Points

**Question:**

What is the expected size of measles outbreaks in Texas at current (ie, 2018) and decreased vaccination rates?

**Findings:**

In this decision analytical model study of metropolitan statistical areas in Texas, 1 in 20 measles introductions was forecasted to be associated with outbreaks of more than 400 cases in the Austin and Dallas metropolitan areas, according to 1000 simulated outbreaks in each metropolitan area; 64% of cases were forecasted to occur in children for whom a vaccine has been refused and 36% in others (ie, bystanders). Increased vaccine exemptions were forecasted to be associated with increases in the expected size of measles outbreaks.

**Meaning:**

The 2018 vaccination rates in multiple metropolitan areas may permit large measles outbreaks, which could infect not only vaccine refusers but also other members of the population.

## Teaser

This study uses a decision analytical model to simulate measles outbreaks in metropolitan areas in Texas using 2018 and future reduced vaccination rates.

## Introduction

Measles is a highly infectious disease that can result in severe health outcomes, including pneumonia, brain damage, and death.^1^ Immunity to measles can be gained through vaccination. In the United States, the Centers for Disease Control and Prevention recommends that children receive a first vaccination dose at ages 12 to 15 months and a second dose at ages 4 to 6 years (before school entry), although some children are not vaccinated because of contraindications.^2^ Vaccination does not confer immunity after 2 doses in 3% of people.^2^ Approximately 92% to 96% of a homogeneously mixed population requires immunity to achieve herd immunity,^3^ although vaccination coverage requirements should be adjusted for local demographic conditions.^4^

Measles is no longer endemic in the United States,^5^ but regular outbreaks still occur when individuals exposed in other countries enter the United States,^6,7^ causing tens to hundreds of cases annually in recent years.^8^ The 3 largest outbreaks since elimination infected hundreds of people each,^9,10,11^ the last of which led to mandatory vaccinations ordered in New York, New York.^12^

Efforts to achieve or maintain herd immunity have been hampered by a small segment of the population declining vaccinations for their children for various reasons, including concerns regarding adverse effects of vaccination, lack of knowledge of the vaccine, and social influences.^13,14^ These concerns are not homogeneously spread in the population, creating spatial foci with greater risk of measles outbreaks.^15,16^ Because of these fears increasing in the past 2 decades, vaccine declination disproportionately affects children. A 5% decrease in vaccination rates has been estimated to cause a tripling of measles cases in children aged 2 to 11 years.^17^

Schools with a large number of students with vaccine exemptions provide environments in which measles can spread among susceptible students and to the wider population.^18^ The merits of requiring children to be vaccinated to attend schools have been debated extensively,^19^ with requirements and incentives varying around the world.^20^ In the United States, states individually choose whether to grant vaccination exemptions to allow unvaccinated children to attend school. As of June 2019, exemptions for religious or personal reasons are permissible in 45 states.^21^

Since 2003, the number of reported conscientious exemptions (including personal and religious exemptions) among Texas students has continuously increased from 2300 to 64 000 (by a factor of 28),^22,23^ raising concerns about the risks of future measles outbreaks in Texas.^22,24^ Measles outbreaks in Texas metropolitan statistical areas (MSAs) between 2006 and 2017 involved a median (interquartile range) of 2.5 (1-3) cases, with the largest outbreak involving 25 cases.^25^ An association has been found previously between vaccine exemption laws and incidences of preventable diseases, and Texas’s vaccine exemption laws are among the least effective at reducing vaccine exemptions in the United States.^26^ In 2009 to 2017 in Texas, 60% of measles cases occurred in children younger than 19 years.^25^

Texas is the second largest US state by population. It encompasses 24 MSAs (discounting Texarkana, which straddles the Texas-Arkansas state border) with a range of population sizes reflecting 92% of the MSAs in the United States.^27^ By the metric of population size, Texas MSAs can be considered representative of many US population centers. In the United States, an MSA is a geographic area comprising at least 1 urban area of at least 50 000 people and the surrounding counties from which people commute to the urban areas.^28^ Texas MSAs include population sizes both greater than and less than the critical community size (300 000-500 000) at which epidemic irregularity and demographic stochasticity (random variability in mortality and birth rate) are important components of measles outbreaks.^29,30^

We use agent-based simulations of the Texas population, including the size, location, and vaccination rate of each school, to evaluate the risk of widespread measles outbreaks occurring within and beyond Texas schools. We analyze the risk of outbreaks at 2018 vaccination rates (from the 2017-2018 school year) and the risk if vaccination rates continue to decrease. Advocacy groups in Texas have argued both in favor of and against changes to vaccine exemption regulations in Texas^31,32,33^; this investigation aims to help inform such discussions. Early (unpublished) versions of these simulations directly influenced legislative votes in favor of the elimination of conscientious exemptions to vaccination in California.^34^ Simulations were run for each Texas MSA (except Texarkana). Texas counties not located in an MSA are not discussed in this article; however, the results of similar simulations are provided elsewhere.^35^

## Methods

### Agent-Based Model

An agent-based decision analytical model was created using the Framework for Reconstructing Epidemiological Dynamics (FRED) tool.^36^ Agent-based models simulate the actions and interactions of a collection of individual agents that follow a set of rules. By use of an agent-based model, the behavior of agents in a complex, interconnected system can be studied.^37^ The FRED tool can model the spread of infectious diseases within a population. The daily interactions of agents in their household, neighborhood, and school or workplace (if the agent is set to attend a school or workplace) are simulated, allowing a contagion to spread.

The agents in these simulations were characterized with a synthetic population of Texas.^38,39^ The population was generated from 2010 US Census data to accurately represent the demographic distribution of each US Census tract in Texas, including age, gender, race, household size, and household income. Each member of the synthetic population was assigned a household location according to US Census tract populations and demographic distributions. Agents were assigned workplaces corresponding to commuting patterns, business sizes, and employment rates.^38,39^

Each school-aged agent was assigned a school type (public or private) and school location on the basis of their demographic characteristics.^38,39^ The schools in the synthetic population were created using real school locations and sizes. The agent-based model assumes that, during each work day, agents interact with the other agents in their household, neighborhood, and workplace or school.

The basic reproduction number of measles, *R*_0_, has previously been found^40,41^ to be 12 to 18 (although it can vary over larger ranges^42^). Agents’ daily number of contacts was calibrated to reflect the accepted transmission rates for measles, with a median *R*_0_ of 13.3 (interquartile range, 12.0-14.7) (eAppendix 1 in the Supplement). The simulations’ *R*_0_ range does not include some of the higher estimates, so these simulation results may underestimate the overall spread of measles outbreaks. Whether a potentially infectious contact results in a new infection depends on the susceptibility of the receiving agent; immunized agents do not become infected.

The relative number of contacts in each location was calibrated using estimates that, during infectious disease outbreaks in a completely susceptible population, 30% of all transmissions occur in households, 33% occur in the neighborhood, and 37% occur in schools and workplaces. The per capita rate of transmissions in schools is double that in workplaces.^43,44^

 All data used in this study were publicly available and therefore did not require approval from an institutional review board (according to 45 CFR part 46). This study follows the non–cost-related Consolidated Health Economic Evaluation Reporting Standards (CHEERS) reporting guideline.^45^

### Vaccination Rates

Student vaccination rates were assigned on the basis of the reported vaccination rate of the equivalent real school that agents attended. The 2-dose measles, mumps, and rubella vaccination is 97% effective against measles^2^; therefore, 3% of vaccinated students were randomly assigned as susceptible to measles. Approximately 0.2% of students in Texas are ineligible to be vaccinated because of contraindications^46^; medical exemptions to vaccination were assigned randomly to agents in schools.

People born in the United States before 1957 are assumed to be immune to measles because they were exposed before mass vaccination, when measles was widespread.^47^ Agents representing population members aged 62 years or older (the age of someone born before 1957 on January 1, 2019) were, thus, immune to measles in these simulations. The vaccination rate of the remaining population was assumed to be 94.8%, on the basis of antibody seroprevalence analysis of the US population^48^ and assuming 97% effectiveness of administered vaccinations.

Vaccination rates for each school were obtained from the Texas Department of State Health Services.^23^ The most recent available vaccination rate for each school was used (from the 2017-2018 to 2015-2016 school years). Vaccination rates for schools with no data available were estimated using the vaccination rates of similar, nearby schools (eAppendix 2 and eFigure 1 in the Supplement). Private schools in Texas report vaccination rates on a per-school basis; however, public schools report by school district, so the vaccination rate of a school district was applied to each public school in the district. Seventh-grade (ages 12-13 years) vaccination rates were used (eAppendix 3 in the Supplement).

### Measles Model

In each simulation run, a single case of measles was introduced via a randomly selected student (aged 5-15 years) for whom a vaccine had been refused. Measles outbreaks were simulated for the 2018 vaccination rates at each school, and further scenarios in which the vaccination rate of schools with vaccine refusers was decreased by 1% to 10%. Approximately 0.2% of students in Texas are reported as being medically exempt from vaccination. We assume an uncertainty of 0.2% on this value; therefore, any school with less than 99.6% of students vaccinated was considered to have vaccine refusers when deciding which schools’ vaccination rates to decrease.

Decreasing the vaccination rate in schools with undervaccinated populations allowed investigation of the potential outcomes of vaccination exemptions becoming more commonplace. Schools that already have students for whom vaccines have been refused may represent the schools at greatest risk of higher rates of vaccination refusal in the future.

One thousand simulations were run for each vaccination rate decrement in each Texas MSA. Measles outbreaks were simulated for 270 days (ie, 9 months), and the number of cases was counted. No interventions or changes in behavior (eg, mass vaccination campaigns or restricting school access for unvaccinated children) were modeled. Thus, the purpose of this model is to estimate the risk of possible outbreaks, not to predict the results of possible public health interventions. Interventions for measles outbreaks have previously been investigated with an early version of FRED.^49^

### Statistical Analysis

The FRED models are stochastic simulations in which outcomes vary according to the interactions of particular individuals. In some simulations, the randomly selected agent who serves as the primary case of measles may have limited daily contacts with susceptible agents (ie, an agent with both a small household and school size) and no outbreak may arise. In other runs, the primary case may have a different set of contacts that leads to a major outbreak. Because our purpose is to estimate the potential risk of a major outbreak, we have chosen to report the number of cases at the 95th percentile from 1000 simulation runs in each MSA. This is the upper range of the number of cases that are forecast to occur with at least 5% probability and, therefore, have a significant risk of occurring from an initial single measles infection. Simulations were run with the FRED software.^36^ Analysis was performed with the Python programming language version 2.7 (Python Software Foundation), using the NumPy,^50^ Scikit-learn,^51^ SciPy,^52^ Pandas,^53^ and Seaborn^54^ libraries.

## Results

**Figure 1.**
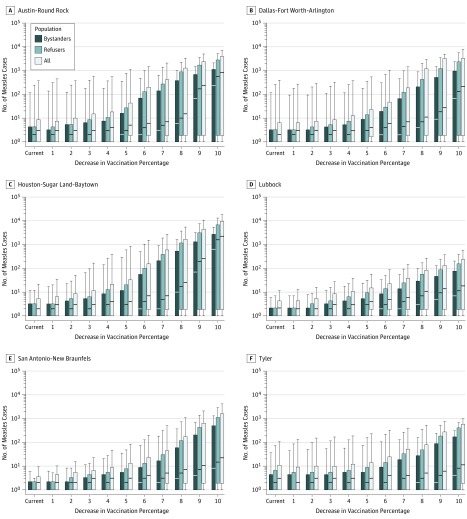
Forecasted Number of Cases From a Single Introduction of Measles Graphs show forecasted number of measles cases from a single introduction by a student for whom a vaccine has been refused, at 2018 and reduced vaccination rates. The number of cases among bystanders, refusers, and the combined population are plotted. Vaccination rates are decreased only in schools that currently have students for whom measles vaccination has been refused. Results for 6 different metropolitan statistical areas in Texas are shown. The other metropolitan statistical areas are plotted in eFigure 2 in the Supplement. Each bar shows the median (the horizontal line within each bar) and interquartile range (top and bottom of each bar); whiskers show the 5% to 95% confidence interval.

Figure 1 shows the expected number of measles cases in 6 MSAs, including the 4 largest MSAs in Texas (Austin–Round Rock, Dallas–Fort Worth–Arlington, Houston–The Woodlands–Sugar Land, and San Antonio) and 2 with more typical population sizes (Lubbock and Tyler); the population estimates used for each MSA are provided in the eTable in the Supplement. The total number of cases is split between refusers and bystanders, where refusers are students for whom a vaccine has been refused for nonmedical reasons and bystanders are other susceptible members of the population (comprising those for whom vaccination failed, students who are medically exempt, and nonstudents who are not vaccinated).

Among all simulations using 2018 vaccination rates, where 25 or more agents became infected, a mean (SD) of 36% (11%) of cases occurred in bystanders, and a mean (SD) of 64% (11%) of cases occurred in students for whom a vaccine had been refused. Simulations that produced outbreaks with 3 to 24 infections had a larger mean, but with a greater variability (mean [SD], 49% [24%]) in the percentage of bystander cases.

In our simulations, large outbreaks occurred in some MSAs at 2018 vaccination rates with at least 5% probability. Although the median number of cases from each outbreak ranged from 1 to 3, there was a significant chance (at least 1 in 20 measles introductions) of large outbreaks in 3 MSAs: more than 400 cases in Austin–Round Rock and Dallas–Fort Worth–Arlington (the 95th percentile, associated with 1 in 20 measles introductions) and more than 100 cases in Tyler (associated with 2 schools with vaccination rates of 70% and 85%). In the remaining MSAs, an outbreak was likely to be more limited, with a mean (SD) 95th percentile of 12 (6) measles cases.

**Figure 2.**
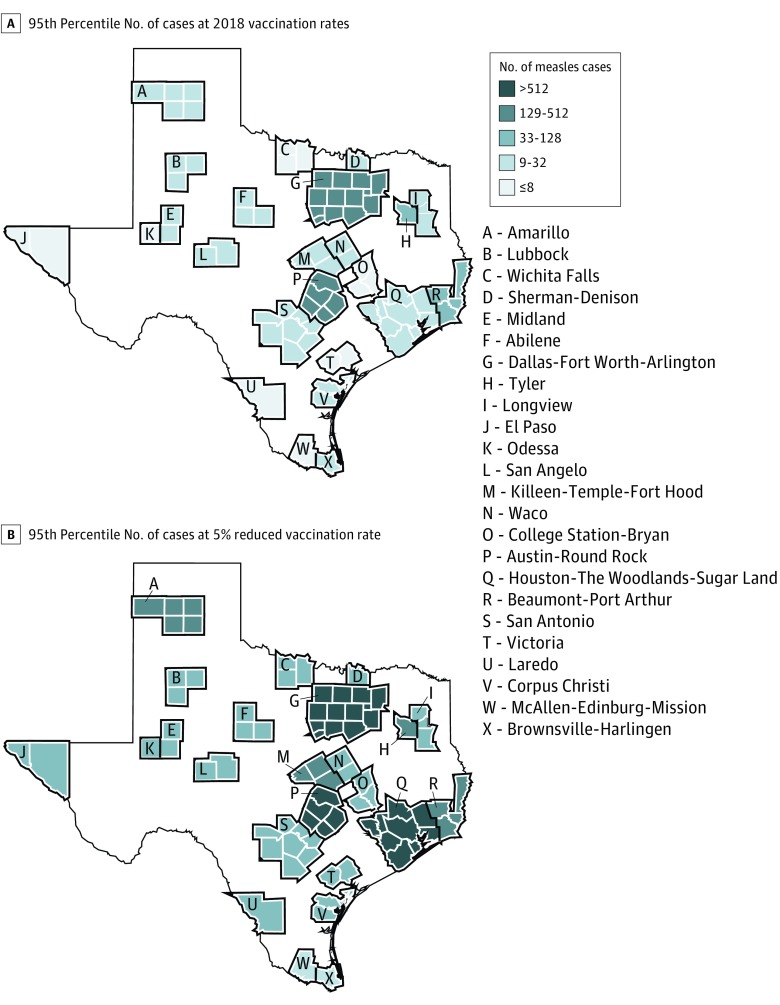
Map of Forecasted Number of Measles Cases in Each Metropolitan Statistical Area of Texas Forecasted numbers of measles cases in each metropolitan statistical area, resulting from a single introduction by a student for whom a vaccine has been refused, are shown. Maps show the 95th percentile number of cases at 2018 vaccination rates (A) and if the vaccination rate at schools with currently undervaccinated populations were to decrease by 5% (B). Metropolitan statistical area boundaries are shown in black, and county boundaries are shown in white.

Figure 1 shows the expected number of cases if the vaccination rate were to decrease in schools with populations that are currently undervaccinated. The expected numbers of cases in the other MSAs are plotted in eFigure 2 and briefly discussed in eAppendix 4 in the Supplement. A map showing the numbers of measles cases forecast at the 95th percentile of cases, for both 2018 and decreased vaccination rates, is displayed in Figure 2.

Decreasing the vaccination rate in schools with undervaccinated populations resulted in an approximately exponential increase in the total number of cases and the number of bystander cases at the 95th percentile (eAppendix 5 and eFigure 3 in the Supplement). A 5% decrease in vaccination rate was associated with a 40% to 4000% increase in potential outbreak size, depending on the MSA. The median number of cases in each MSA, however, remained low for small reductions in vaccination rates. Larger reductions in vaccination rates were associated with rapid increases in the median number of cases for a single outbreak: an 8% reduction led to median outbreak sizes of at least 10 cases in 25% of MSAs, whereas a 9% reduction led to median outbreak sizes of at least 10 cases in 71% of MSAs.

## Discussion

These simulations estimate that large measles outbreaks of more than 400 cases could occur in Austin–Round Rock and Dallas–Fort Worth–Arlington, with their 2018 distribution of vaccination rates. This finding is consistent with the largest measles outbreaks that have occurred since measles was eliminated in the United States^9,10^ and suggests that the vaccination rate of these areas should be increased to reduce the chance of a large outbreak.

The Tyler MSA simulations suggest a significant chance of large measles outbreaks associated with students attending 2 schools with vaccination rates of 70% and 85% (eAppendix 6 in the Supplement). This highlights that a small number of significantly undervaccinated networks could be associated with measles spreading widely in a population. In the event of an outbreak in schools with undervaccinated populations, interventions targeted at these schools may be especially effective. Such interventions may involve isolation measures or mandatory vaccinations, as happened in New York State in 2019,^12^ or voluntary vaccination programs,^18^ if vaccination acceptance increases during an outbreak. Undervaccinated close-knit communities present an increased risk of outbreaks; mandating that schools with low vaccination rates plan for outbreak scenarios may help reduce outbreak sizes.

The large number of cases that could occur in Austin–Round Rock, Dallas–Fort Worth–Arlington, and Tyler corroborates concerns previously expressed about the decreasing vaccination rate in parts of Texas.^22^ The potential for large outbreaks is partially associated with the large populations in these MSAs, but also with the presence of schools with undervaccinated populations in these areas: there are 35, 13, and 15 schools, respectively, in these MSAs with vaccination rates less than 92% (the lower band of the estimated herd immunity threshold).^22^

A relatively high potential for outbreaks in Austin and Dallas, compared with elsewhere in the United States, has previously been found.^55^ The forecast distributions in outbreak sizes are consistent with recent and ongoing outbreaks across the United States: approximately one-half of US measles cases imported from abroad from January 1, 2019, to April 26, 2019, resulted in no secondary cases,^56^ but there have been larger outbreaks of 71, 275, and 609 cases (the latter 2 are ongoing) in Washington and New York States.^10,11,57^ Seventeen outbreaks (defined as ≥3 cases) occurred in the United States in 2018,^58^ compared with a median of 4 outbreaks per year from 2001 to 2012.^59^

The simulations suggest that large outbreaks occur where there is a significant population of students for whom a vaccine has been refused; however, infections are not limited to those students. More than one-third of cases may affect individuals whose vaccination has failed, who are unable to be vaccinated, or who are unvaccinated nonstudents (who are at greater risk of complications due to measles than are children aged 5-19 years).^60^

The median number of cases in most of the MSAs ranged from 1 to 3. This is because of many instances when the initial case is seeded in an agent who has few interactions with susceptible agents, such as students who attend highly vaccinated schools or schools with small enrollments. The probability of each infectious agent interacting with susceptible agents is significantly increased in undervaccinated networks, schools in particular.

Decreasing the vaccination rates of schools with currently undervaccinated populations is associated with an increase in the number of cases at the upper confidence limit in an approximately exponential manner, which is similar to previous estimations.^17^ The rate of increase in the number of cases varies between MSAs; this may be associated with differences in the size and number of schools with currently undervaccinated populations in each MSA. Nonmedical exemptions in Texas schools have annually increased since 2003,^22,23^ suggesting that further decreases in vaccination rates are likely without a change in public perception or policy. Large measles outbreaks may be associated with such a change in public perception but can also result in severe health outcomes for those infected.

Lower vaccination rates imply that outbreaks may occur with greater frequency, because there are both more people who can become exposed to measles when away from their MSA and more people who can be infected by an exposed individual from elsewhere. In addition, refusers may be locally grouped, sharing schools and communities, creating a greater risk of measles introductions spreading to a large number of unvaccinated students.

### Limitations

The model’s assumptions should be considered when interpreting these simulation results. Texas public school districts only publish the vaccination rate of the district, preventing the simulations from revealing the risk associated with specific public schools with low vaccination rates. If data for individual public schools were published, heterogeneities in the risk of large measles outbreaks could be investigated more accurately. Legislation has been proposed to allow these data to be published,^31^ but concerns have been expressed regarding privacy.^33^ In addition, ensuring that all schools and school districts report their vaccination rates to the Texas Department of State Health Services (which is mandated by law but not complied with universally) could better allow spatial distributions in risk to be analyzed.

We assume that the seventh-grade vaccination rates provide a more accurate representation of schoolwide vaccination rates than kindergarten vaccination rates, because we assume that unvaccinated kindergarten students who are delinquent, provisionally enrolled, or using a delayed vaccination schedule will receive vaccinations in a timely manner. If they do not, the potential size of outbreaks may be larger than forecasted here.

The measles outbreaks are simulated in each MSA in isolation. Although human interaction within each MSA is greater internally than with other MSAs, a large outbreak in any MSA may lead to cases spread beyond its borders. This may increase the number of cases caused by each measles outbreak above the numbers simulated here.

The relative ratios of human interactions in schools, workplaces, neighborhoods, and households are based on previous studies^43,44^; however, these ratios likely vary from community to community. Changes to these ratios may affect forecasted outbreaks; for example, a greater fraction of interactions occurring in schools may lead to greater outbreak sizes. Differences in the interactions of vaccine-hesitant communities may be especially influential in measles transmission. In addition, this model does not include mass gatherings, such as sporting events or theme parks, which may facilitate measles transmission.

The daily interactions of the population of Texas and transmission of measles within it are part of a large, complex system. Data pertaining to the synthetic population and measles transmission must be estimated to model it. The US Census and federal databases^38,39^ were used to generate the synthetic population, and the transmissibility of measles was calibrated to published values of the basic reproduction number of measles.^40,41^ However, it is possible that other synthetic populations and parameter values may be estimated using alternative methods and data sources, which may yield differing forecasts.

The initial exposed agent in each simulation is selected from students for whom a vaccination has been refused. This means it is more likely that the initial infection occurs in a school with an undervaccinated population than from randomly selecting from all susceptible population members, thus increasing the chances of secondary infections. This seeding method was used because this study focuses on measles infections associated with refused vaccinations in schoolchildren.

Simulations that forecast outbreak sizes with decreased vaccination rates do not include an estimate as to when these decreases in vaccination rates may occur. The population and school sizes may change over time, which may affect simulation forecasts. Furthermore, the fraction of adults born before 1957 (who are assumed to be immune) will decrease with time, potentially creating a more susceptible population.

## Conclusions

Simulations of potential measles outbreaks in Texas, using 2018 school vaccination rates, suggest that there is a significant chance of outbreaks totaling more than 100 cases in multiple geographic areas. If the vaccination rate among students in Texas continues to decrease in schools with undervaccinated populations, the potential number of cases associated with measles outbreaks is estimated to increase exponentially. It is estimated that in measles outbreaks with more than 25 cases, a mean (SD) of 36% (11%) of cases may occur in people who are not students for whom a vaccine has been refused.

## Notes

Supplement. **eAppendix 1.** Measles Model **eAppendix 2.** Kernel Density Estimation **eFigure 1. **Probability Density Function (Blue Line) of Vaccination Rates of Schools in Lubbock County, Texas **eAppendix 3.** Vaccination Data **eAppendix 4.** Vaccination Decrease **eAppendix 5.** Vaccination Decrease: Fits **eAppendix 6.** Synthetic Population **eTable.** Population Sizes Used in Simulations **eReferences** **eFigure 2. **Forecast Number of Measles Cases at Current and Reduced Vaccination Rates **eFigure 3. **Exponential Fits to the Expected Number of Measles Cases in Each Metropolitan Statistical Area
