# Supplementary material for: Forecasted Size of Measles Outbreaks Associated With Vaccination Exemptions for Schoolchildren
Source: JAMA Netw Open. 2019 Aug 21;2(8):e199768. doi: 10.1001/jamanetworkopen.2019.9768 (PMC6707017; doi:10.1001/jamanetworkopen.2019.9768)
Supplement: Supplement. — eAppendix 1. Measles Model eAppendix 2. Kernel Density Estimation eFigure 1. Probability Density Function (Blue Line) of Vaccination Rates of Schools in Lubbock County, Texas eAppendix 3. Vaccination Data eAppendix 4. Vaccination Decrease eAppendix 5. Vaccination Decrease: Fits eAppendix 6. Synthetic Population eTable. Population Sizes Used in Simulations eReferences eFigure 2. Forecast Number of Measles Cases at Current and Reduced Vaccination Rates eFigure 3. Exponential Fits to the Expected Number of Measles Cases in Each Metropolitan Statistical Area [file jamanetwopen-2-e199768-s001.pdf]

Supplementary Online Content

Sinclair DR, Grefenstette JJ, Krauland MG, et al. Forecasted size of measles outbreaks associated with vaccination exemptions for schoolchildren. *JAMA Netw Open*. 2019;2(8):e199768. doi:10.1001/jamanetworkopen.2019.9768

eAppendix 1. Measles Model
eAppendix 2. Kernel Density Estimation
eFigure 1. Probability Density Function (Blue Line) of Vaccination Rates of Schools in Lubbock County, Texas
eAppendix 3. Vaccination Data
eAppendix 4. Vaccination Decrease
eAppendix 5. Vaccination Decrease: Fits
eAppendix 6. Synthetic Population
eTable. Population Sizes Used in Simulations
eReferences
eFigure 2. Forecast Number of Measles Cases at Current and Reduced Vaccination Rates
eFigure 3. Exponential Fits to the Expected Number of Measles Cases in Each Metropolitan Statistical Area

This supplementary material has been provided by the authors to give readers additional information about their work.

© 2019 Sinclair DR et al. *JAMA Network Open.*

eAppendix 1. Measles Model

The basic reproduction number, R_0, is the expected number of new infections caused by a single infectious person in a completely susceptible population. It is reported as 12-18 for measles [5,6]. FRED simulations are calibrated to produce a specified R_0 by adjusting the probability that an interaction between an infectious agent and a susceptible agent results in the susceptible agent becoming infected.

Iterating over different values of this transmissibility parameter gave a best fit of 13.3 (12.0-14.7), from 100 simulations of a completely susceptible Austin-Round Rock MSA population. In each calibration simulation, 100 school students were randomly selected to seed an outbreak, and the average number of secondary infections caused by these 100 agents used to calculate the R_0 for the transmissibility parameter value. 100 initial cases were used to ensure a representative distribution in the number of secondary infections, however this may have resulted in a slight decrease in the measured value of R_0 if agents were competing for the same secondary infections.

eAppendix 2. Kernel Density Estimation

5.1% of the schools in the agent-based model were unable to be matched to a real school's reported vaccination rate. Primarily this was due to a minority of individual private schools and public school districts surveyed by the Texas Department of State Health Services not reporting their vaccination rates (17% in 2017/2018, 12% in 2016/2017 [1,2]. Public school districts more reliably reported their vaccination rates than individual private schools; as each public school district represents multiple schools, the overall percentage of unmatched schools was only 5.1%. Additional factors include that a minority of schools providing their address as a Post Office (PO) box, rather than their street address, when supplying vaccination data. Furthermore, some schools may have opened, closed or moved address from when the school data used in the synthetic population was collected and when the vaccination data was collected. In some instances, this made it unfeasible to match vaccination data to a synthetic school.

It was necessary to infer the vaccination rates of schools without reported vaccination data. The probability density function of the vaccination rates of the schools in each county was estimated using kernel density estimation with machine learning on the reported vaccination rates. A grid search leave-one-out cross-validation was used to optimize the bandwidth of each kernel density estimation. This was performed with scikit-learn's GridSearchCV algorithm [3]. A vaccination rate for each unmatched school was then drawn from the probability distribution of that school's county and type (i.e. public or private). With the combined matched and inferred data, the simulations used an overall student vaccination rate of 98.9%; this is consistent with the overall reported vaccination rate of seventh graders in Texas in 2017-18 [1].

It is recommended that at least four data samples be used when performing a kernel density estimation [4]. If at least four vaccination rates in a county for a public or private school were not available, reported vaccination rates from both types of schools were used for kernel density estimation. If the number of reported vaccination rates available was still fewer than four, a kernel density estimation was not performed. Instead a reported vaccination rate for a school in the county was randomly drawn and used instead.

eFigure 1 shows a sample distribution of school vaccination rates in Lubbock County, Texas. The reported vaccination rate of the schools in the county are shown, alongside the estimated vaccination rates of four schools. The corresponding probability density function was generated using a kernel density estimation on the reported data.

eFigure 1. Probability Density Function (Blue Line) of Vaccination Rates of Schools in Lubbock County, Texas

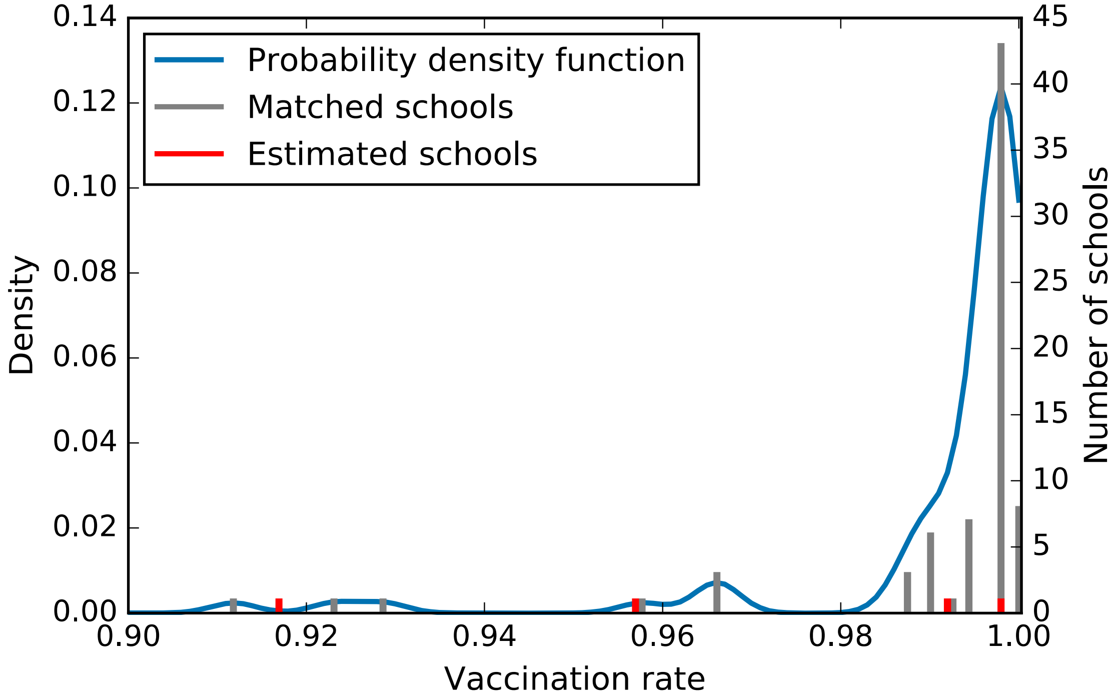

Gray bars indicate the reported vaccination rate of schools in the county which are matched to schools in the FRED simulation. The red bars indicate the estimated vaccination rate of four schools, which were unmatched to reported vaccination rates, drawn from the probability density function.

Measles was modeled with several disease stages. An agent exposed to measles took 11.5 ± 1.23 days (median and dispersion of a lognormal distribution) to enter the 'latent' stage. After one day in the latent state, agents spent three days in the 'fever' stage, before entering the 'rash' stage for four days. A recovery period of 10 days followed, before the agent acquired immunity [7]. Agents were infectious during the latent, fever and rash stages. Agents had a 50% chance of household confinement during the fever stage and 95% during the rash stage.

The measles outbreaks were simulated for 270 simulation days. This approximates the length of the school year. The mixing behavior of children may differ during the school summer vacation.

eAppendix 3. Vaccination Data

Texas records the two-dose vaccination rate of MMR, at kindergarten and grade seven. The recorded kindergarten vaccination rates are typically lower than the seventh grade rates. This is due to provisional enrollments and delinquencies (data is collected early in the school year) as well as increased personal exemption rates among students in kindergarten [1]. A proportion of provisionally enrolled or delinquent kindergarten students may have received a single MMR dose (which is recommended to be administered at 12-15 months [8]) but were late in receiving their second dose (which is recommended to be administered at 4-6 years, before school entry [8]) rather than having parents who intend to forgo vaccination altogether. Delays in receiving a second MMR dose may be due to accidental tardiness, or intentionally delaying vaccination doses beyond the recommended times [9]. As a single MMR dose confers immunity to measles in 93% of recipients [8], these students are at less risk of contracting measles than students who have received no MMR doses.

© 2019 Sinclair DR et al. *JAMA Network Open.*

For the purposes of this study it is assumed that the provisional enrollments and delinquencies are addressed in a timely manner, as Texas law requires these students to be excluded from schools until they are vaccinated or receive an exemption. We assume that when parents intentionally delay vaccination, the delay period is short compared to the period between kindergarten and seventh grade [10] (although this may overestimate the overall vaccination rate). The seventh grade vaccination rates are therefore assumed to provide a more accurate representation of each school's overall vaccination rate than the kindergarten rates.

Approximately 2.2% of students in the synthetic population are designated as home-schooled, in accordance with census data [11]. Home-schooled students were assumed to be vaccinated at the same rate as the background population, 94.8%. In some US states with stricter vaccination requirements, parents who do not wish to vaccinate their children may be required to home-school, potentially lowering the vaccination rate amongst home-schooled students. However, as Texas allows vaccination exemptions, home-school vaccination rates are assumed not to differ significantly from the mean population level. As home-schooled students can be assumed to interact with a small number of other students compared to students who attend schools, the impact of home-schooled students on the spread of an epidemic is expected to be less than that of other students.

eAppendix 4. Vaccination Decrease

eFigure 2 shows the distribution of the number of measles cases that could occur in the metropolitan statistical areas (MSAs) of Texas (not including the MSAs shown in the main document). Each plot shows the number of cases caused by the seeding of an outbreak in a student for whom a vaccination has been refused. Figure e2 also shows how the expected number of cases increases as the vaccination rate in currently under-vaccinated schools decreases from 1-10%.

There is an apparent slight decrease in cases in Dallas-Fort Worth-Arlington for small decreases in vaccination rate (eFigure 2h). This suggests that more samples are required to more precisely determine the upper confidence limit of forecast cases in Dallas-Fort Worth-Arlington. This is because Dallas-Forth-Worth-Arlington has a population of 6.4 million (the fourth largest MSA in the US) and 1303 under-vaccinated schools; depending on the school in which an outbreak commences, there is variation in the expected overall size of an outbreak. Further simulations are beyond the scope of the current investigation as they would require significant computing resources.

eAppendix 5. Vaccination Decrease: Fits

Figure e3 shows fits to the upper confidence limit of the number of measles cases in each MSA. Exponential fits describe the increase in cases for reduced vaccination rates. Differences in the fits between MSAs are due to population sizes and the number of students attending under-vaccinated schools. As vaccination rates were decreased only in currently under-vaccinated schools, the number and size of these schools had a significant effect on the rate at which measles cases increased.

eAppendix 6. Synthetic Population

The agent population size in each metropolitan statistical area (MSA) used in the simulations is given in the eTable. Population sizes are based on the 2010 US census [12] and the Office of Management and Budget's 2015 designations of MSAs [13].

© 2019 Sinclair DR et al. *JAMA Network Open.*

eTable. Population Sizes Used in Simulations

MSA	Population	MSA	Population
Abilene	163 633	Longview	212 824
Amarillo	252 207	Lubbock	289 186
Austin-Round Rock	1 707 666	McAllen-Edinburg-Mission	768 693
Beaumont-Port Arthur	401 771	Midland	140 717
Brownsville-Harlingen	405 040	Odessa	136 325
College Station-Bryan	228 446	San Angelo	110 926
Corpus Christi	425 181	San Antonio-New Braunfels	2 134 062
Dallas-Fort Worth-Arlington	6 408 159	Sherman-Denison	120 740
El Paso	799 891	Tyler	208 975
Houston-The Woodlands-Sugar Land	5 886 884	Victoria	93 411
Killeen-Temple	404 030	Waco	250 694
Laredo	249 691	Wichita Falls	151 044

Agents selected to attend a public school were assigned to the one of the three closest public schools in their county. Students selected to attend a private school were assigned the closest private school to their household without regard for county boundaries [11,14]. Assuming students attend a school near their home may not be true for students attending religious or philosophically-orientated private schools. Hence the locations of infections transmitted from students in these schools to their family members and neighbors may be concentrated into a smaller geographical area than reality.

The model assumes that all medical-exempt children are vulnerable to measles, but some children may have received a medical exemption after previously contracting measles, which would lead to a higher immunity rate than estimated. We also assume medical-exempt students attend schools; severely immuno-compromised children may be more likely to be home-schooled to lessen their risk of infections. However, as medical-exempt students make up a small percent (0.2%) of the student population, these assumptions are unlikely to have a significant effect on the simulation results.

Neighborhoods were defined using a 1 km grid imposed on the household locations.

Agents who attend a school, attend their school on weekdays only. Most workers attend their workplaces on weekdays, however 20% are designated as weekend workers. Neighborhood contacts are increased by 50% on weekends to reflect weekend schedules of schools and workplaces.

The Tyler MSA has large potential outbreak sizes in part due to two schools with vaccination rates of 70% and 85%. It should be noted that one of these schools is a private school located in the adjacent Longview MSA. The algorithm used to assign students to private schools, which is based on distance and not county boundaries, assigned a significant number of students who live in the Tyler MSA to this school (which lies between the two MSAs' urban cores). The outbreak risk may thus be overestimated in the Tyler MSA and underestimated in the Longview MSA.

eReferences

[1] Texas Department of State Health Services, 2017-2018 results of annual report of immunization status, Online, available at https://www.dshs.texas.gov/immunize/coverage/schools. Accessed August 2018, 2018.
[2] Texas Department of State Health Services, 2016-2017 results of annual report of immunization status, Online, available at https://www.dshs.texas.gov/immunize/coverage/schools. Accessed November 2018.
[3] Pedregosa F., Varoquaux G., Gramfort A., et al., Scikit-learn: Machine Learning in Python, J Mach Learn Res, 2011, 12, 2825–2830.
[4] Silverman B., Density Estimation for Statistics and Data Analysis, Chapman & Hall/CRC Monographs on Statistics & Applied Probability, Taylor & Francis, 1986.
[5] Anderson R., May R., Directly transmitted infections diseases: control by vaccination, Science, 1982, 215, No. 4536, 1053–1060.
[6] Anderson R.M., May R.M., Age-related changes in the rate of disease transmission: implications for the design of vaccination programmes, J Hyg (Lond), 1985, 94, No. 3, 365–436.

© 2019 Sinclair DR et al. *JAMA Network Open.*

[7] J. H., Kroger A., Wolfe S. (Eds.), Centers for Disease Control and Prevention. Epidemiology and Prevention of Vaccine-Preventable Diseases, chap. Measles, Public Health Foundation, Washington D.C., 13 edn., 2015.
[8] McLean H.Q., Fiebelkorn A.P., Temte J.L., Wallace Gregory S., Prevention of Measles, Rubella, Congenital Rubella Syndrome, and Mumps, 2013: Summary recommendation of the Advisory Committee on Immunization Practices (ACIP), MMWR Morb Mortal Wkly Rep, 2013, 62(RR04), 1–34.
[9] Offit P.A., Moser C.A., The problem with Dr Bob's alternative vaccine schedule, Pediatrics, 2009, 123, No. 1, e164–e169.
[10] Smith P.J., Humiston S.G., Marcuse E.K., et al., Parental delay or refusal of vaccine doses, childhood vaccination coverage at 24 months of age, and the Health Belief Model, Public Health Reports, 2011, 126, No. 2 suppl, 135–146.
[11] Wheaton W.D., Cajka J.C., Chasteen B.M., Wagener D.K., Cooley P.C., Ganapathi L., Roberts D.J., Allpress J.L., Synthesized Population Databases: A US Geospatial Database for Agent-Based Models, Methods report (RTI Press), 2009, , No. 10, 905–905, doi:10.3768/rtipress.2009.mr.0010.0905, URL http://www.ncbi.nlm. nih.gov/pmc/articles/PMC2875687/.
[12] United States Census Bureau, Annual estimates of the resident population: April 1, 2010 to July 1, 2017, Online, available at https://factfinder.census.gov/faces/tableservices/jsf/pages/productview. xhtml?pid=PEP_2017_PEPANNRES&prodType=table. Accessed October 2018, 2018.
[13] Executive Office of the President, Office of Management and Budget, Washington D. C. 20503, OMB Bulletin No. 15-01, 2015.
[14] RTI International, U.S. Synthetic population 2010 version 1.0 quick start guide, Online, available at http://www. epimodels.org/10_Midas_Docs/SynthPop/2010_synth_pop_ver1_quickstart.pdf, 2014.

© 2019 Sinclair DR et al. *JAMA Network Open.*

eFigure 2. Forecast Number of Measles Cases at Current and Reduced Vaccination Rates

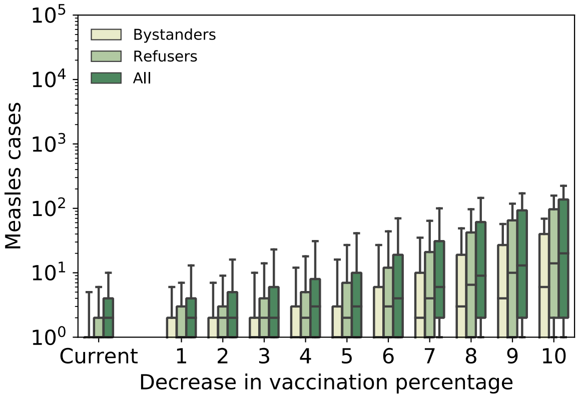

(a) Abilene

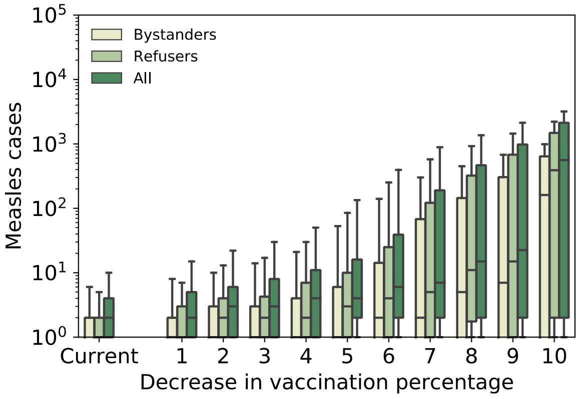

(b) Amarillo

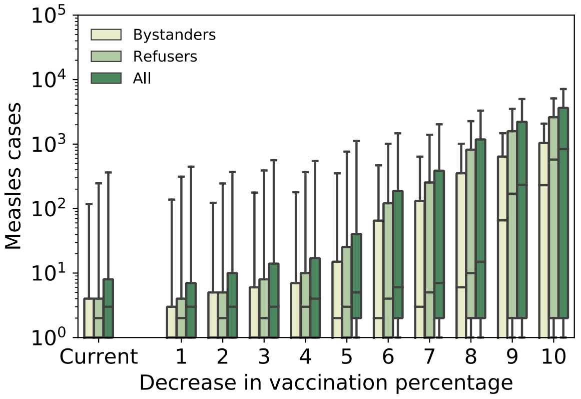

(c) Austin-Round Rock

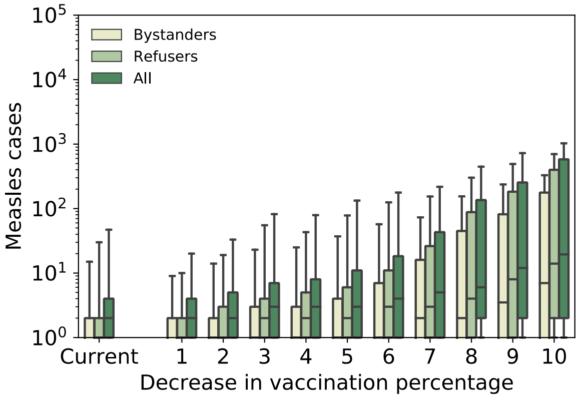

(d) Beaumont Port Arthur

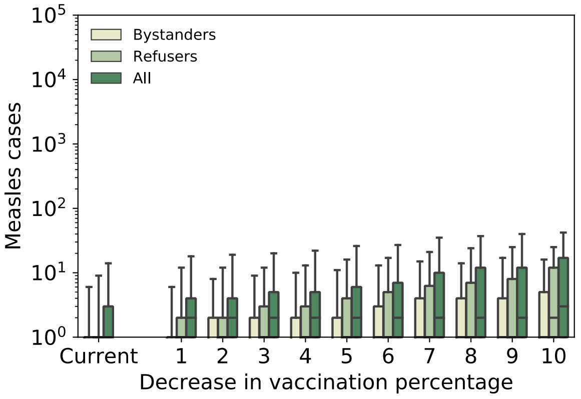

(e) Brownsville-Harlingen

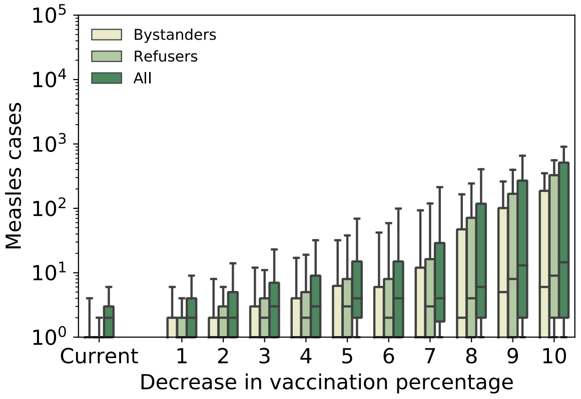

(f) College Station-Bryan

© 2019 Sinclair DR et al. *JAMA Network Open.*

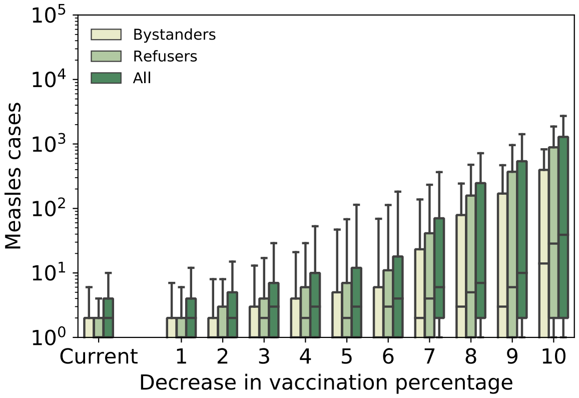

(g) Corpus Christi

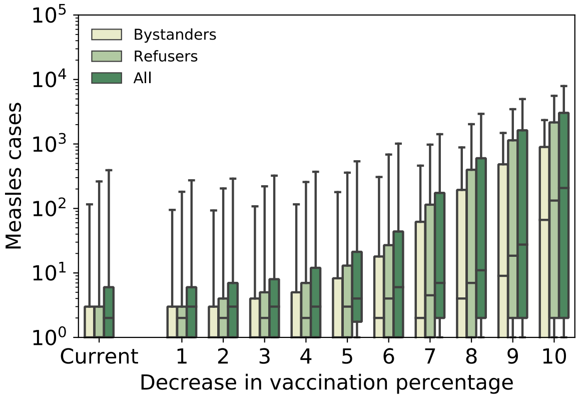

(h) Dallas-Fort Worth Arlington

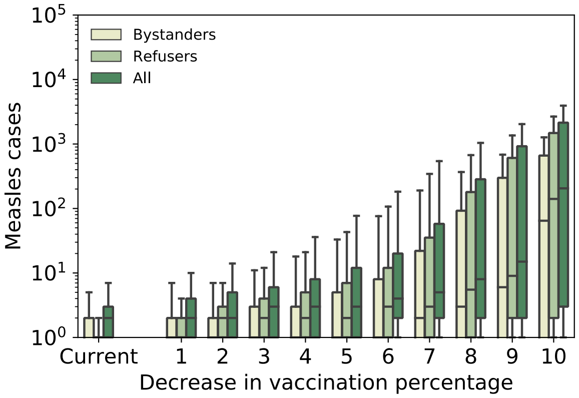

(i) El Paso

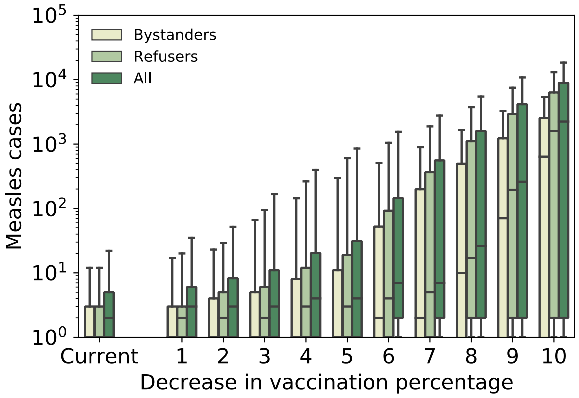

(j) Houston-The Woodlands-Sugar Land

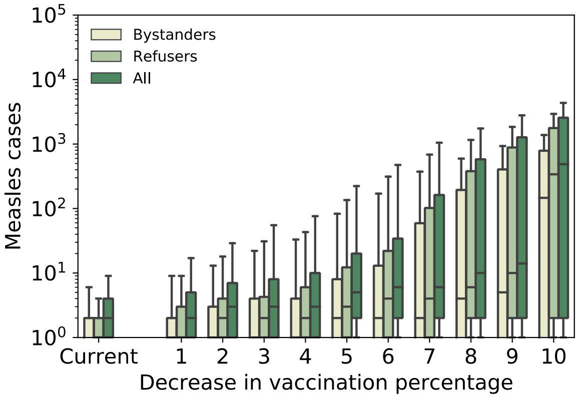

(k) Killeen-Temple-Fort Hood

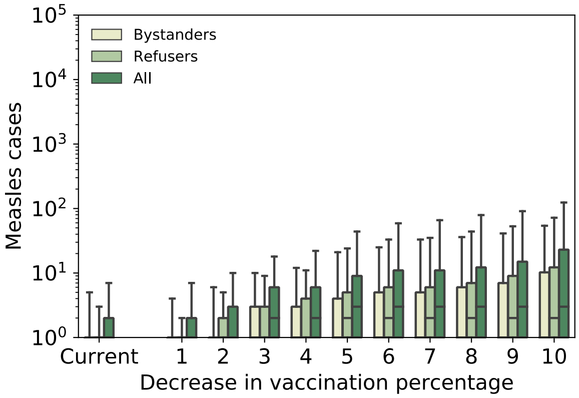

(l) Laredo

© 2019 Sinclair DR et al. *JAMA Network Open.*

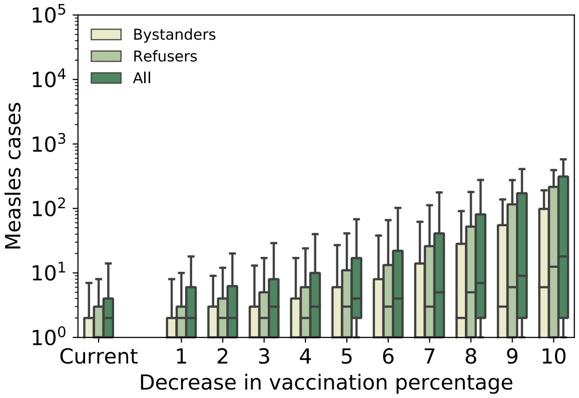

(m) Longview

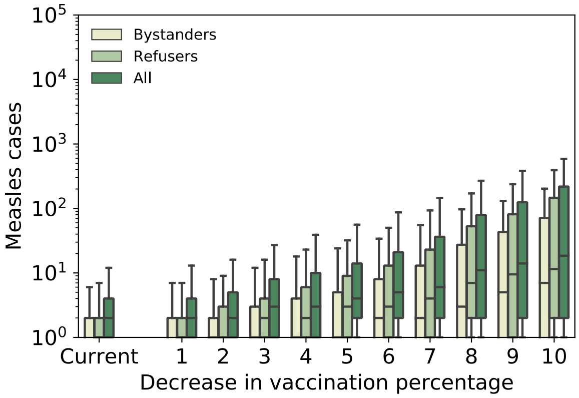

(n) Lubbock

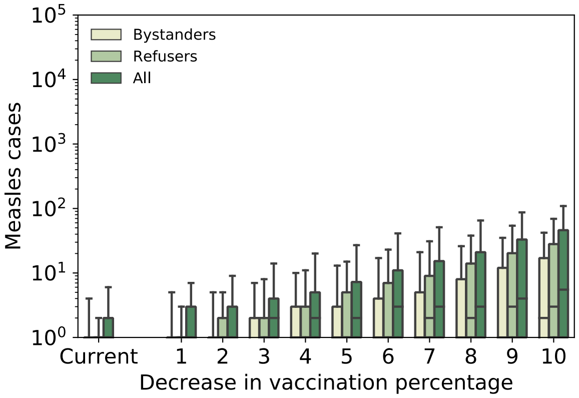

(o) McAllen-Edinburg-Mission

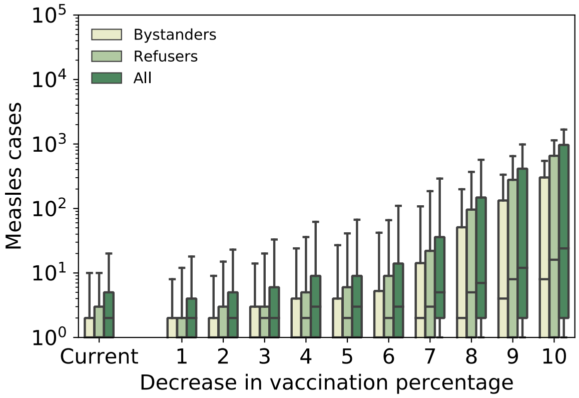

(p) Midland

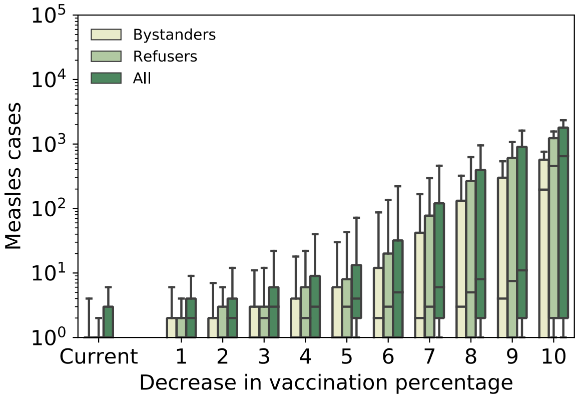

(q) Odessa

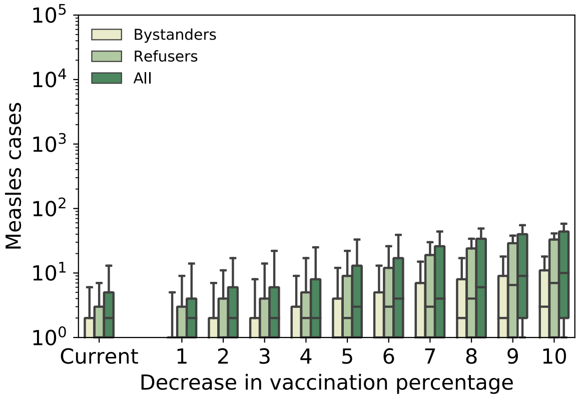

(r) San Angelo

© 2019 Sinclair DR et al. *JAMA Network Open.*

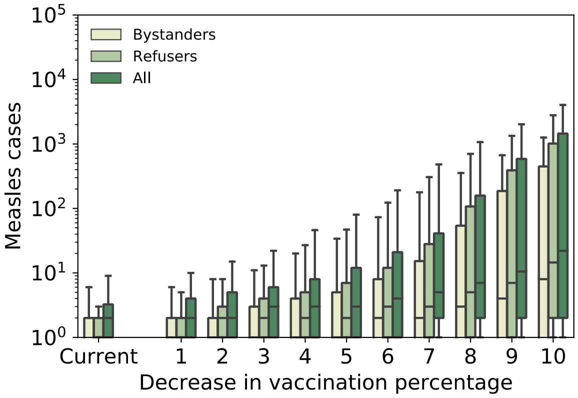

(s) San Antonio

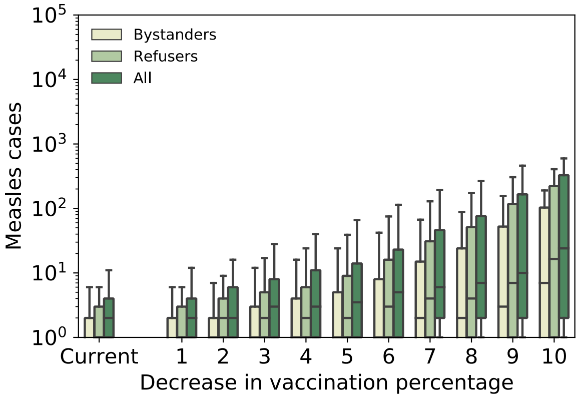

(t) Sherman-Denison

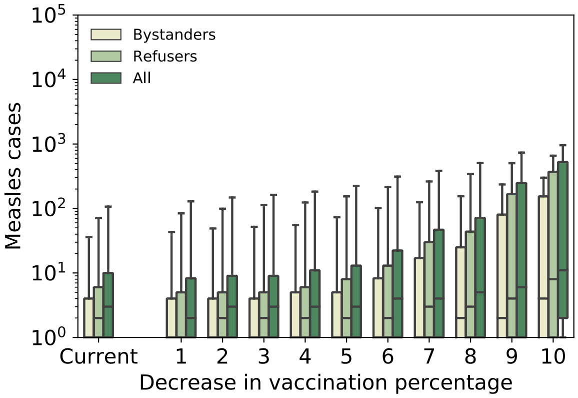

(u) Tyler

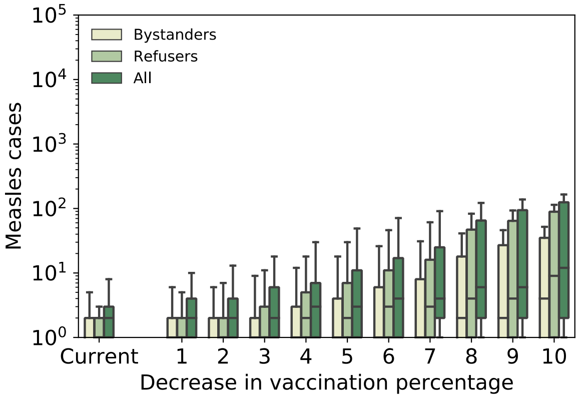

(v) Victoria

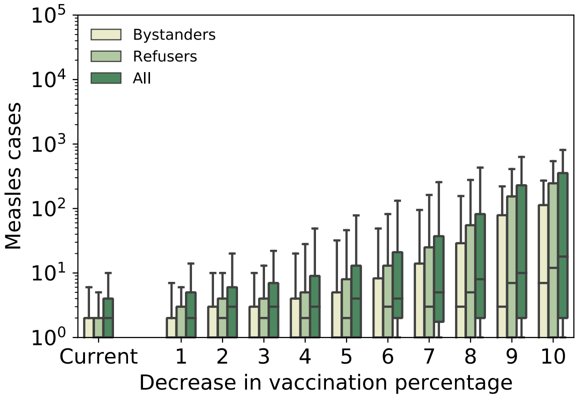

(w) Waco

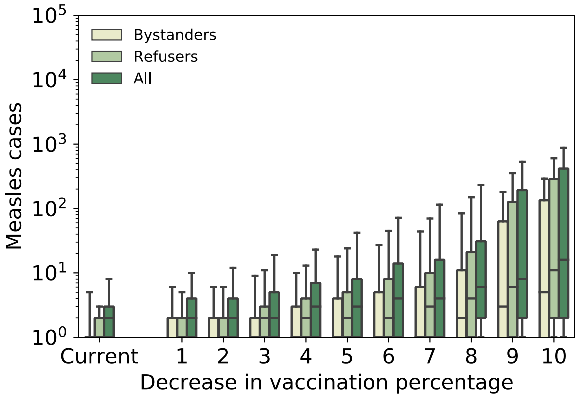

(x) Wichita Falls

Forecast number of cases from an initial infectious student, for whom a vaccine has been refused, at current and reduced vaccination rates. The number of cases among bystanders, refusers and the combined population are plotted. Vaccination rates are only decreased in schools which currently have students for whom measles vaccination has been refused. Results for the metropolitan statistical areas in Texas not included in fig. 2 of the main document are shown. Each bar shows the median and interquartile range; whiskers show the 5-95% confidence interval.

© 2019 Sinclair DR et al. *JAMA Network Open.*

eFigure 3. Exponential Fits to the Expected Number of Measles Cases in Each Metropolitan Statistical Area

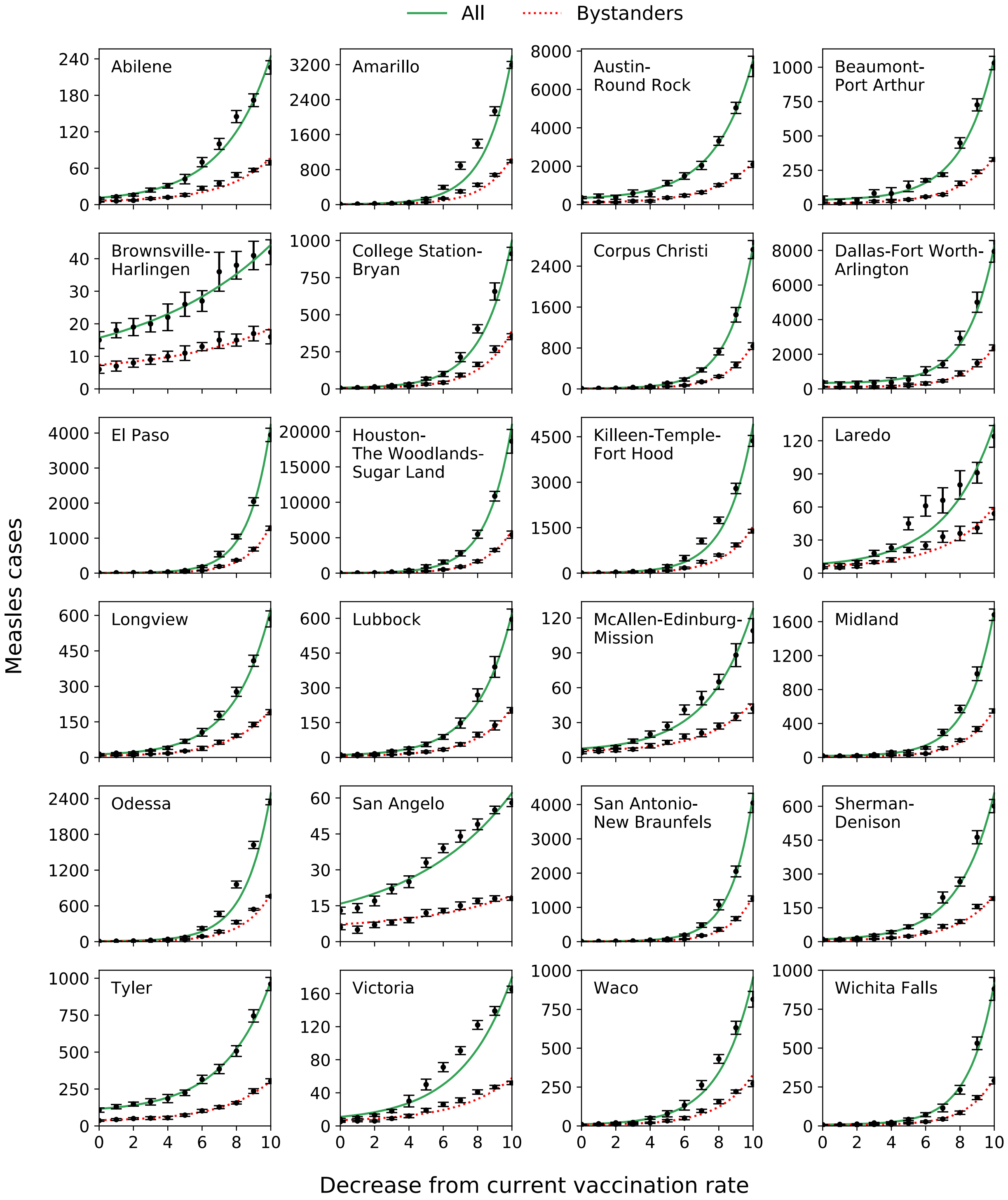

Exponential fits to the 95th percentile of number of the expected number of measles cases in each Metropolitan Statistical Area. Fits are shown for all cases, and cases among bystanders only. Uncertainties on 95th percentile calculated using a nonparametric bootstrap on the simulated data (1000 samples, each of size 1000). Fits are of the form $y = Ae^{Bx} + C$, where A; B and C are free parameters.

© 2019 Sinclair DR et al. *JAMA Network Open.*

© 2019 Sinclair DR et al. *JAMA Network Open.*
